# Dissolved carbon storage and flux dynamics in China's inland waters over the past 30 years

**DOI:** 10.1093/nsr/nwaf229

**Published:** 2025-06-02

**Authors:** Shuoyue Wang, Gaboury Benoit, Peter A Raymond, Guirui Yu, Feng Zhou, Shaoda Liu, Chiyuan Miao, Kun Sun, Zhaoxi Li, Junjie Jia, Yang Gao

**Affiliations:** Key Laboratory of Ecosystem Network Observation and Modeling, Institute of Geographic Sciences and Natural Resources Research, Chinese Academy of Sciences, Beijing 100101, China; College of Resources and Environment, University of Chinese Academy of Sciences, Beijing 100049, China; Yale School of the Environment, Yale University, New Haven, CT 06511, USA; Yale School of the Environment, Yale University, New Haven, CT 06511, USA; Yale School of the Environment, Yale University, New Haven, CT 06511, USA; Key Laboratory of Ecosystem Network Observation and Modeling, Institute of Geographic Sciences and Natural Resources Research, Chinese Academy of Sciences, Beijing 100101, China; College of Resources and Environment, University of Chinese Academy of Sciences, Beijing 100049, China; Institute of Carbon Neutrality, Laboratory for Earth Surface Processes, College of Urban and Environmental Sciences, Peking University, Beijing 100871, China; Key Laboratory of Water and Sediment Sciences of Ministry of Education and State Key Laboratory of Water Environment Simulation, School of Environment, Beijing Normal University, Beijing 100875, China; State Key Laboratory of Earth Surface Processes and Resource Ecology, Faculty of Geographical Science, Beijing Normal University, Beijing 100875, China; Key Laboratory of Ecosystem Network Observation and Modeling, Institute of Geographic Sciences and Natural Resources Research, Chinese Academy of Sciences, Beijing 100101, China; State Key Laboratory of Hydroscience and Engineering, Tsinghua University, Beijing 100084, China; Key Laboratory of Ecosystem Network Observation and Modeling, Institute of Geographic Sciences and Natural Resources Research, Chinese Academy of Sciences, Beijing 100101, China; College of Resources and Environment, University of Chinese Academy of Sciences, Beijing 100049, China; Key Laboratory of Ecosystem Network Observation and Modeling, Institute of Geographic Sciences and Natural Resources Research, Chinese Academy of Sciences, Beijing 100101, China; College of Resources and Environment, University of Chinese Academy of Sciences, Beijing 100049, China

**Keywords:** inland waters, dissolved carbon pattern, aquatic carbon cycle, driving mechanisms

## Abstract

Inland waters (lakes, reservoirs, and rivers) serve as important regulators of global climate change and carbon (C) cycling. China's inland water systems significantly regulate regional C budgets. However, our understanding of the long-term spatiotemporal patterns and underlying mechanisms of dissolved carbon (DC) storages and fluxes in inland waters remains limited. This study examined lake and reservoir DC storage and river DC flux, quantifying their changes in China over the past three decades. We found that inland water DC stocks in China increased from 96 Tg C in the 1990s to 142 Tg C in the 2010s while DC river flux did not significantly change (13.2 ± 0.4 Tg C/yr). Findings also showed that a combination of climate change, anthropogenic disturbance, and water chemistry collectively drove inland water DC dynamics. River DC was more directly driven by climate and anthropogenic factors (>50%) while lakes and reservoirs were more directly influenced by water chemistry (>70%). Additionally, climate factors can explain changes in dissolved inorganic carbon (DIC) concentrations via water chemistry factors (i.e. electrical conductivity (EC) and pH), while, collectively, climate and the nutrient status can typically explain changes in dissolved organic carbon (DOC) concentrations. This study emphasizes the important role that inland water plays in the global C balance and underscores the necessity of considering it in future C budgets.

## Teaser

Over the past three decades, inland waters in China have significantly increased their dissolved carbon storage, with distinct drivers for lakes, reservoirs, and rivers, revealing their critical role in global carbon cycling.

## INTRODUCTION

A central theme of the global carbon (C) cycle and climate change research is the ‘missing carbon sinks’. Many of these missing C sinks abide within terrestrial ecosystems [1]. Among these terrestrial ecosystems, inland water bodies (lakes, reservoirs, rivers, and streams) play a critical yet often overlooked role in global C cycling and climate change. This is because they exchange carbon dioxide (CO_2_) with the atmosphere and connect the two largest C reservoirs, namely, terrestrial and marine ecosystems. Despite covering only 2.7% of the Earth's non-glaciated continental surface [2], inland waters emit a disproportionate amount of greenhouse gas (GHG) to the atmosphere [3,4]. Globally, it has been estimated that inland waters emit ∼3.9 Pg C yr^−1^ of CO_2_ and CH_4_ to the atmosphere, offsetting the contemporary terrestrial CO_2_ sink (2.3 Pg C yr^−1^) [5,6]. Annually, global rivers transport ∼5.1 Pg C yr^−1^ from land to inland waters while further transporting 0.9 Pg C yr^−1^ into estuaries [6]. Inland waters transport, bury, and remove C before it reaches coastal ocean regions, thereby significantly altering the global C budget [7]. Compared with other ecosystems, inland water bodies possess extremely efficient ecosystem C cycling and C sequestration functions with a high C sequestration potential [8]. However, inland water C sink assessments are not currently included in the global C budget [5], consequently increasing uncertainties in reconciling regional carbon flux discrepancies, climate feedbacks, and land–ocean carbon coupling. Accordingly, clarifying long-term spatiotemporal inland water C stock patterns will be highly significant when exploring the ‘missing C sinks’ while simultaneously achieving C neutrality goals.

The main inland water C forms are dissolved organic carbon (DOC), dissolved inorganic carbon (DIC), and particulate organic carbon (POC) [9], of which dissolved carbon (DC) generally accounts for most of the total C within the water column [10]. Through biological or photochemical processes, DOC mineralization has been shown to be a major CO_2_ emission source [9–11]. Additionally, C-based substances will consume dissolved oxygen through peroxide decomposition and water quality deterioration, impacting phytoplankton growth through light absorption [3,6]. Various inland water types and different C forms respond dissimilarly to environmental change. Changes in long-term dissolved C exports have been shown to be significantly regulated by air temperature [12], while hydrological conditions may explain their associated short-term variations [13]. Moreover, DIC is also influenced by CO_2_ evasion, which is also affected by climate and land-use change [14]. Additionally, both climate change and anthropogenic disturbances can impact aquatic biogeochemical processes, directly or indirectly driving inland water DC dynamics. Therefore, exploring the long-term C dynamics and associated driving mechanisms of various inland water body types is crucial for understanding their associated C cycling and CO_2_ emission processes, for developing C reduction strategies, and for achieving C neutrality policy goals.

**Figure 1.**
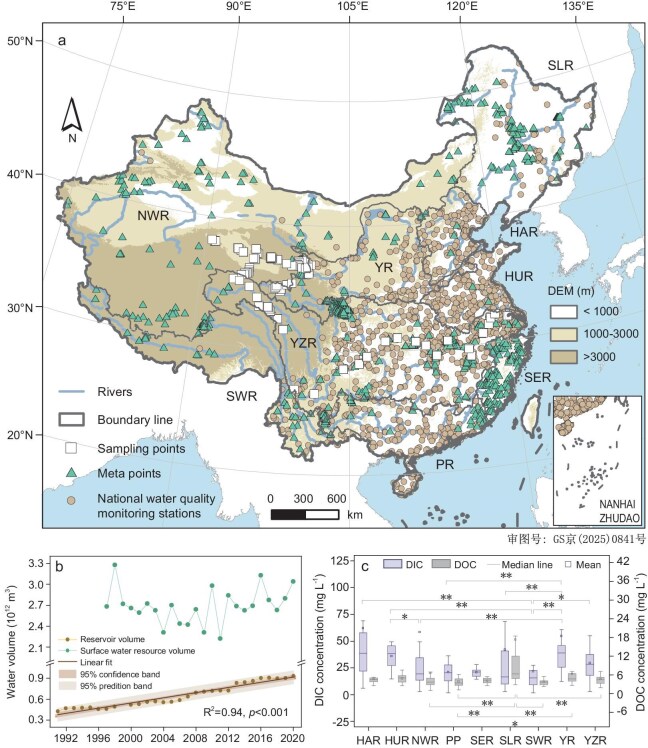
(a) Sampled lakes, reservoirs, and rivers distribution across the nine large drainage catchments in China (*n* = 2455), (b) changes in surface water resources and reservoir capacity in China over the past 30 years, and (c) differences in DC concentrations in nine of China's major watersheds. SLR, the Songhua River and Liaohe River Basin; HAR, the Haihe River Basin; YR, the Yellow River Basin; HUR, the Huaihe River Basin; YZR, the Yangtze River Basin; SER, the Southeast River Basin; PR, the Pearl River Basin; SWR, the Southwest River Basin; NWR, the Northwest River Basin. ** *P* < 0.01; * *P* < 0.05.

Despite occupying only ∼7% of global terrestrial area, China contains critical freshwater systems, accounting for over half of global reservoir capacity, major river basins (e.g. Yangtze and Yellow Rivers), and lakes experiencing rapid transformations driven by anthropogenic and climatic pressures [14,15]. A few studies have previously assessed inland water C stocks and C flux in China's inland waters [16,17]. However, due to the difficulty in obtaining long-term data and spatiotemporal variability, considerable uncertainty remains in our understanding of the long-term spatiotemporal patterns and the underlying mechanisms of inland water DC stocks. In recent decades, China has undergone notable environmental transformations and socio-economic growth. Climate change and anthropogenic activities (i.e. agricultural activities, urbanization, industrial pollution, etc.) can affect the terrestrial ecological environment, which in turn affects the inland water volume and C dynamics (Fig. 1), but their effects have generally remained unexplored [10,12]. The long-term climate and human disturbances in inland waters have complex impacts on carbon dynamics in water bodies through direct effects on watershed carbon transport and indirect effects on water chemistry and nutrients. The impact mechanisms of different types of inland water bodies may vary. The objective of this study is to assess inland water DC storage and flux in China while exploring the underlying mechanisms that have been driving these dynamics over the past three decades. Findings from this study have significant implications for comprehending the global C cycle, balancing inland water C budgets, and effectively managing water quality.

## RESULTS

### Inland water volume and runoff in China over the past 30 years

#### Lake and reservoir volume

**Figure 2.**
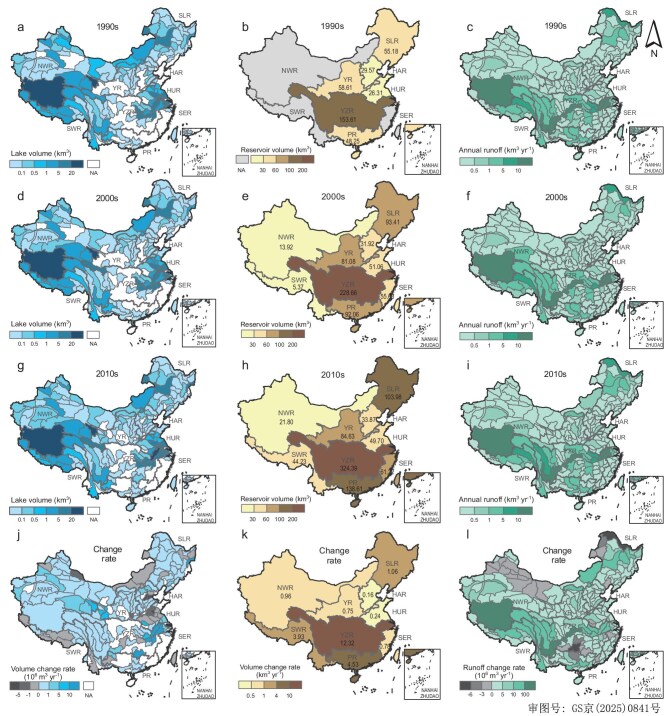
Water quantity spatial patterns of China's inland lakes (≥1 km^2^) during the 1990s (a), 2000s (d), and 2010s (g) and the rate of change between 1990 and 2020 (j); China's spatial reservoir patterns during the 1990s (b), 2000s (e), and 2010s (h) and the rate of change between 2006 and 2020 (k); China's spatial average annual river runoff patterns during the 1990s (c), 2000s (f), and 2010s (i) and the rate of change between 1991 and 2018 (l).

The total natural lake area (≥1 km^2^) in China expanded from 68 000 km^2^ in 1990 to 82 900 km^2^ in 2020 while its total water volume also increased by 51.8 km^3^ (21.9%), where both its spatial distribution and change factors varied (Fig. 2). Approximately half of China's natural lake systems are located in its northwestern river basins, being especially concentrated in the Northwest River Basin (NWR), with a total surface area and total water volume of 51 400 km^2^ and 181 km^3^ in the 2010s, respectively (Fig. 2). Second, natural lake area and water volume in the Yangtze River Basin (YZR) are both considerable (i.e. 34 600 km^2^ and 151 km^3^ in the 2010s, respectively) (Fig. 2). Conversely, natural lake water volume in the Southeast River Basin (SER), the Haihe River Basin (HAR), and the Pearl River Basin (PR) is relatively small, all being <1 km^3^ (Fig. 2). Over the past 30 years, the lake water volume in most basins has shown an increasing trend, with YZR exhibiting the highest rate of change (0.71 km^3^/yr), followed by SLR (0.1 km^3^/yr) and NWR (0.03 km^3^/yr). Specifically, lakes in the middle and lower Yangtze River Plains, Xinjiang, the Northeast Plain, and the Qinghai–Tibet Plateau have significantly increased in water volume, which can be attributed to glacier melting, permafrost thawing, and increased precipitation. Meanwhile lakes in the Huanghuai Plain, the Inner Mongolia Plateau, and the Southwest Mountainous Region have declined in water volume.

Between 1990 and 2020, reservoir construction developed rapidly, for which >15 000 new reservoirs were built, expanding its total capacity from 466 km^3^ to 931 km^3^, nearly doubling the storage capacity of reservoirs (Fig. 1 and Fig. S4). Among them, the construction of reservoirs in southern China has grown rapidly, while in northern China it has been relatively slow. The change rate of reservoir capacity in YZR is the highest, reaching 12.3 km^3^/yr, followed by PR (4.5 km^3^/yr) and SWR (3.9 km^3^/yr) (Fig. 2). Reservoirs in China are mainly located in the YZR, PR, and SLR basins, with respective storage capacities of 324 km^3^, 139 km^3^, and 104 km^3^ during the 2010s (Fig. 2). Although western China has many large lakes, it has very few reservoirs. The area of the NWR accounts for 35% of China's total area, but the total number of reservoirs is <1% of the country's total (Fig. 2). The sparse reservoir development in western China is attributed to topographic barriers, low population density, ecologically sensitive zones, and prohibitive infrastructure costs. Most large reservoirs are also located in eastern China, and their spatial distribution clearly shows a decreasing trend from east to west (Fig. S4). This is because rapid urbanization, high population density, and water availability in eastern China have accelerated the construction of water facilities, especially large reservoirs (Fig. S4). Although the number of large- and medium-sized reservoirs accounts for <5% of the total number of reservoirs, these large- and medium-sized reservoirs account for most of China's total water storage.

#### River runoff

Over the past 30 years, average annual runoff in the YZR basin has been much higher compared with the other basins, reaching 171 km^3^/yr in the 2010s, followed by the SER, the NWR, and the PR, whose annual runoff ranges from 30 to 60 km^3^/yr (Fig. 2). Although many dams were built in the YZR and the PR, their influence on average annual runoff is limited, likely attributable to dam operations primarily redistributing intra-annual flow rather than altering annual runoff volume. Additionally, average annual runoff in the HAR, the SER, and the SLR is low, namely, only 2.8, 17.1, and 18.9 km^3^/yr, respectively, in the 2010s (Fig. 2). As shown in Fig. 2l, all river basins have shown an increasing trend in runoff in the past three decades, among them, the change rate of river runoff in YZR is the highest, at 1 km^3^/yr, followed by NWR (0.5 km^3^/yr) and YR (0.2 km^3^/yr). There was an obvious increasing trend in annual river runoff in the Qinghai–Tibet Plateau region, where most tertiary watersheds increased at a rate >0.1 km^3^/yr (Fig. 2l). This reflects expansion of the river network and increased flow rates in the region under increased precipitation, snow and ice melt, and permafrost thaw. Additionally, these rivers remain largely unaffected by damming and other anthropogenic disturbances. Besides, annual river runoff in the Xiaoxing’anling Forest District, Daxing’anling Prefecture, Yunnan–Guizhou Plateau, Turpan Depression, and Junggar Basin regions all decreased. Runoff decline in these regions is attributed to coupled climatic (declining precipitation, rising temperatures) and anthropogenic drivers (increased ecological water demand, agricultural intensification), exacerbated by groundwater over-extraction in arid basins and reduced water retention capacity from forest degradation and karst drainage losses.

### China's inland water carbon storage and flux over the past 30 years

#### Lake and reservoir carbon storage

**Figure 3.**
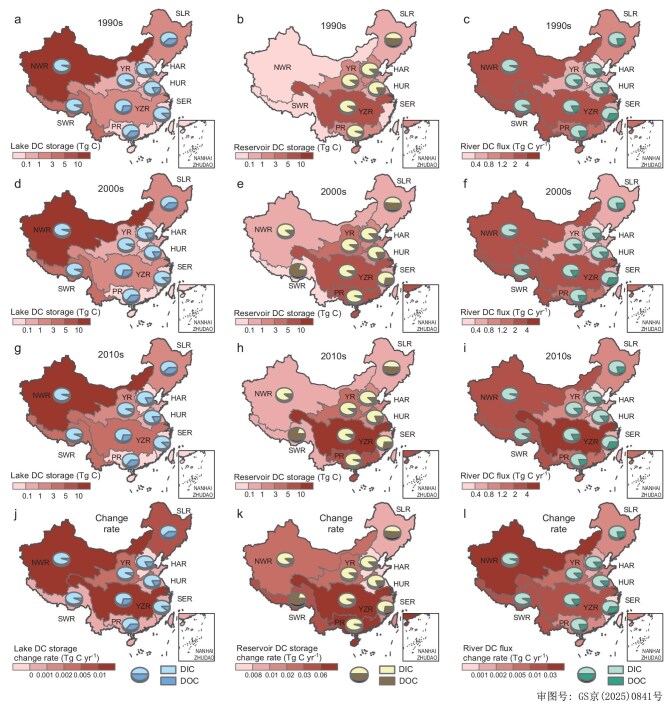
China's lake DC storage spatial patterns during the 1990s (a), 2000s (d), and 2010s (g) and the change rate between 1990 and 2020 (j); China's spatial reservoir DC storage patterns during the 1990s (b), 2000s (e), and 2010s (h) and the change rate between 2006 and 2020 (k); China's spatial river DC flux patterns during the 1990s (c), 2000s (f), and 2010s (i) and the change rate between 1991 and 2018 (l).

As shown in Fig. 3, lake and reservoir DC storage levels within each regional division over the past 30 years were heterogenous. Lake DIC storage was significantly higher in the NWR and SER (84 Tg C and 3 Tg C, respectively) compared with the other basins while lake DOC storage was higher in the NWR, SLR, and YZR (4.1, 0.7, and 0.6 Tg C, respectively) (Fig. 3). The C storage of lakes in the NWR is higher than that of other basins and results from the synergistic effects of their distinctive basin geographies, arid climatic drivers, and natural C accumulation processes under lower anthropogenic disturbance. Additionally, lake DC storage levels were low in the SER and HAR (i.e. both <0.002 Tg C) (Fig. 3). This may primarily be attributed to high hydrological connectivity-driven rapid C export, anthropogenic disturbance-dominated C loss, and the synergistic effects of geochemical processes. In China, lake DC stocks increased from 84 Tg C in the 1990s to 115 Tg C in the 2010s, with an overall respective 36% and 45% increase in DIC and DOC stocks. Reservoir DIC stocks were higher in the YZR (7.2 Tg C) and PR (5.0 Tg C) while they were lower in the NWR and SWR (0.02–0.5 Tg C) due to the lower distribution of reservoirs in these latter regions (Fig. 3). The distribution of reservoirs and catchment vegetation conditions influence the hydraulic retention time and terrestrial C inputs, thereby affecting watershed C stocks. In contrast to DIC storage, reservoir DOC storage in the SWR (0.9 Tg C) was significantly higher compared with the other basins, while the lowest DOC storage level was observed in the NWR (0.07 Tg C) (Fig. 3). Reservoir DC stocks more than doubled due to the rapid increase in reservoir capacity over this period. Additionally, in most primary watersheds, an increasing trend was observed in lake and reservoir DC stocks.

#### River carbon flux

**Figure 4.**
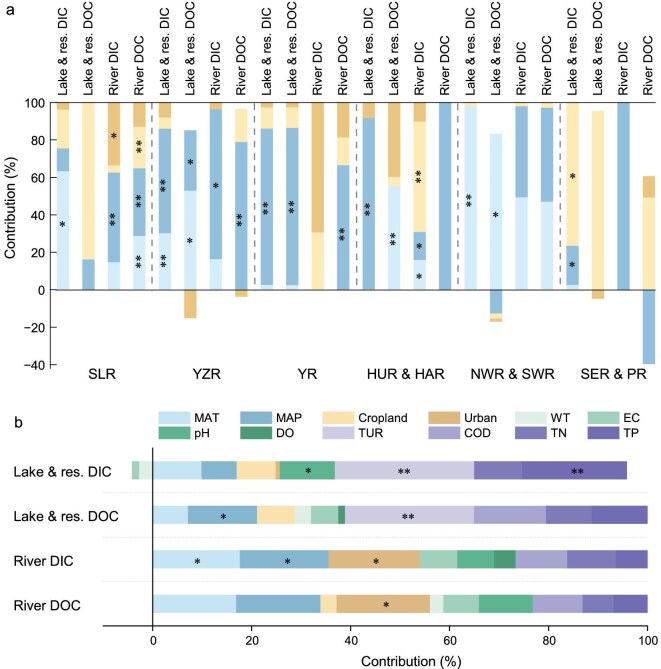
Contribution of climate, land use, and water chemistry to lake reservoir DC stocks and river DC flux. Lake & res. denotes lake and reservoir; MAT, mean annual temperature; MAP, mean annual precipitation; WT, water temperature; DO, dissolved oxygen; EC, electrical conductivity; TUR, turbidity; COD, chemical oxygen demand; TN, total nitrogen; TP, total phosphorus. ** *P* < 0.01, * *P* < 0.05.

**Figure 5.**
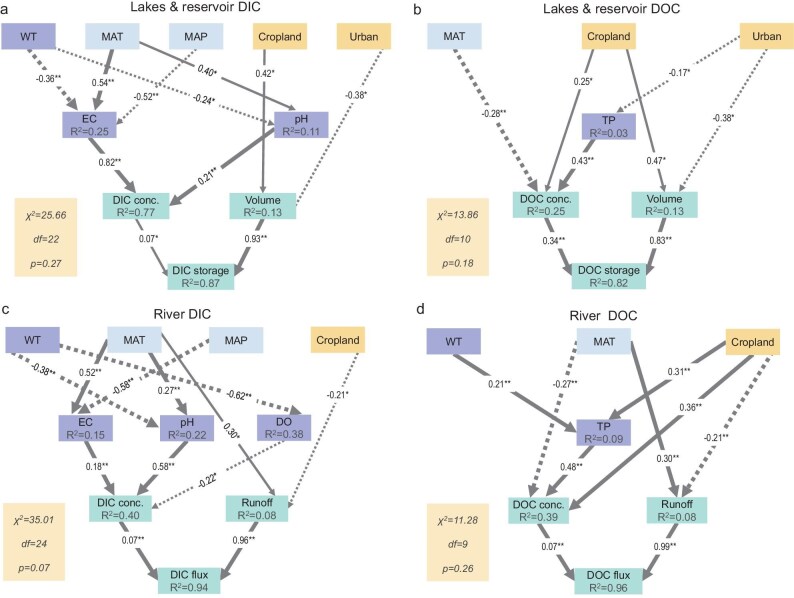
The PLS-SEM model illustrating the impact factors of lake reservoir DC stocks and river DC flux. MAT, mean annual temperature; MAP, mean annual precipitation; WT, water temperature; DO, dissolved oxygen; EC, electrical conductivity; TP, total phosphorus. ** *P* < 0.01, * *P* < 0.05.

As shown in Fig. 3, natural river DIC flux was significantly higher in the YZR, SWR, and NWR compared with the other basins, ranging from 2 to 3.8 Tg C/yr in the 2010s. Similarly, the highest river DOC flux (0.6 Tg C/yr) was observed in the YZR followed by the NWR and SWR, both of which were >0.2 Tg C/yr. Additionally, the HAR and SER regions had the lowest river DIC and DOC flux. Moreover, river DIC and DOC flux was low in the HAR and SER (i.e. 0.11 and 0.02 Tg C/yr in the HAR and 0.12 and 0.07 Tg C/yr in the SER). Overall, DC flux change in China's natural rivers from the 1990s to the 2010s was not significant (i.e. an ∼7% increase) (Fig. 4). Most watersheds exhibited an increasing trend in natural river DC flux, a significantly positive correlation is observed between river carbon fluxes and runoff (*P* < 0.01; Fig. 5). Except for the PR, the highest DC flux observed in all watersheds occurred during the 2010s.

## DISCUSSION

### Impact of climatic and anthropogenic factors 

Climatic factors (temperature and precipitation), anthropogenic factors (agricultural and urban land shares), and water chemistry parameters (water temperature (WT)\EC\pH\dissolved oxygen (DO)\total phosphorus (TP), etc.) jointly drove inland water C dynamics (Fig. S5). Results from this study showed that climate and anthropogenic disturbances were more impactful for river DC flux by affecting hydrological connectivity and terrestrial export, while water chemistry more directly drove lake and reservoir DC stocks (Fig. 4). Factors affecting water chemistry are often synergistic and the degree of interaction varies across spatial scales [19,20].

For China's inland waters, climate and anthropogenic effects on DC stocks and flux have been significant during the Anthropocene. Combined, the variance percentages of river DIC and DOC flux explained by climate and anthropogenic variables were 54% and 56%, respectively, which was far greater than the contribution to lake and reservoir DC stocks (28%–29%) (Fig. 4). Climate warming and anthropogenic disturbances contribute to C accumulation in water bodies by increasing autochthonous and imported allochthonous catchment C content [10]. Generally, the climate tends to have an overwhelming influence on stream chemistry concentrations at a continental scale compared with local watershed characteristics (i.e. topography, lithology, or local material abundance) [21]. Findings showed that climate occasionally did not directly affect DC concentrations at large scales; rather, it indirectly affected DC concentrations through other pathways, such as water chemistry and nutrients. There exist multiple ecological and biogeochemical pathways that exchange energy and nutrients between terrestrial and aquatic environments [22–24]. The influx of large amounts of C from terrestrial to river ecosystems is critical for river ecosystem productivity [25,26], defining the composition and structure of aquatic communities and the cross-subsidization of the terrestrial environment [27,28].

For climatic factors, the impact of mean annual temperature (MAT) and mean annual precipitation (MAP) is relatively large on the DC of lakes, reservoirs, and rivers. The contribution of river DC flux variance explained by MAT and MAP ranges from 16% to 18%, while the contribution of lake and reservoir DC stocks ranges from 7% to 14% (Fig. 4). Interactions between precipitation and temperature drive changes in solute concentrations in different biomes [29,30]. Temperature and precipitation also significantly affect water chemistry factors, which have a significant impact on biogeochemical aquatic processes and consequently water C content (Fig. 5). Changes in DIC concentrations are typically explained by climate through water chemistry factors, such as EC and pH, while changes in DOC concentrations are typically explained by climate through the nutrient status (TP) (Fig. 5). MAT had a significant positive effect on EC, while MAP had a significant negative effect on EC (*P* < 0.01; Fig. 5). EC indicates the concentration of ions in the water column, and higher water temperatures accelerate natural chemical reactions and release excess nutrients into water, resulting in an increase in solute concentrations in the water column. Additionally, a warmer climate may alter the hydrological budget of a catchment, thereby affecting terrestrial C and nutrient transport to lakes [31,32]. Increased precipitation delivers more material to a catchment but also dilutes solute concentrations in water bodies, where the dilution effect of MAP on inland water bodies is more pronounced at a national scale. Precipitation, the permeability, and slope affect the connectivity and flow paths within a catchment [33] and consequently the storage or transport of leachable solutes [34]. Additionally, findings showed that the correlation between water temperature and nutrient status (primarily TP) was significantly positive, which in turn positively affected water DOC concentrations (*P* < 0.01). Warmer temperatures may also cause higher aquatic primary productivity and increase the amount of organic C produced within watersheds [35,36].

Inland water C dynamics observed during the Anthropocene cannot be explained by climate alone. Anthropogenic landscape change also plays a role [37]. Variance in China's lake and reservoir DC stocks can be mainly explained by changes in the proportion (7%–9%) of agricultural land, while river DC flux is mainly influenced by the proportion (18%–19%) of urban land (Fig. 4b). Agricultural, industrial, and residential effluent discharge and nonpoint source pollution increase organic matter inputs from land-based sources within watersheds, thereby affecting both the C dynamics and nutrient status of watersheds [38]. Globally, C emissions from human sewage have been estimated at 100 Tg C/yr, mainly organic [39]. Structural equation modeling results showed that the agricultural land use share can directly and positively affect inland water DOC concentrations while indirectly and positively affecting water DOC levels through its influence on the nutrient status of water bodies (Fig. 5; *P* < 0.05). Wang *et al.* (2023) reported that anthropogenic disturbances related to urbanization and agricultural land use had a great impact on inland water CO_2_ emissions, which was greater than catchment productivity [40]. Intensive agriculture is a major source of soil erosion, leading to the subsequent transport of sediment, terrestrial organic C, and nutrients to inland waters [41]. High-sediment basins enhance sedimentary C burial through substantial POC inputs, yet concurrently intensify C mineralization and release at sediment–water interfaces. In contrast, low-sediment regions rely on DOC-dominated fluxes, where C retention efficiency is modulated by karst hydrological pathways. Mixed cropping and fertilization of arable land, the densification of urban settlements, and industrial development are the main causes that lead to nutrient changes (i.e. nitrogen [N] and phosphorus [P]) in water bodies [42,43]. Consequently, this may directly increase aquatic organic C through heavy transport loads and the efficient conservation of organic C from terrestrial sources and indirectly by stimulating aquatic productivity [44,45]. Therefore, various land use types may reflect diverse anthropogenic activity types. Additionally, anthropogenically-induced landscape alterations may also have a direct impact on the water quantity and flow of inland water bodies (*P* < 0.05; Fig. 5).

At a watershed scale, findings showed that all watershed DC stocks and flux were strongly influenced by climatic factors, except for SER and PR (Fig. 4). Hypothetically, this may be due to the better climatic conditions within the SER and PR regions, which are not limited by temperature and precipitation (Fig. S1). Basin climate conditions varied greatly in this study and were controlled by different climate factors (Fig. S1), where YZR and YR basin DC stock drivers were more reliant on MAP while corresponding NWR and SWR drivers were more reliant on MAT (Fig. 4a). The effect of land use type on water C also varies at different spatial scales. For example, at a national scale, river DIC flux was not directly affected by the agricultural land use share, whereas at a watershed scale, the contribution of agricultural land use to river DIC flux in the HUR and HAR watersheds was highly significant (Fig. 4b; *P* < 0.01). Compared with the other watersheds, the HUR and HAR watersheds had the largest share of agricultural land (Fig. S3), which may subsequently lead to their stronger direct transport of nutrients and fertilizers to streams within their watersheds, thus confirming a different impact compared with a national scale. Economic development and urbanization vary widely from region to region; therefore, the degree of anthropogenic disturbances in water varies widely in different regions (Fig. S2 and Fig. 4a). More economically developed regions are more susceptible to anthropogenic disturbances, with lake DC stocks in the SER and PR basins being more affected by the agricultural land use share and river DIC flux in the YR and SLR basins being more affected by the urban land use share. For example, in the NWR and SWR, both being sparsely populated and economically less developed, the impact of anthropogenic disturbances was very small (<3%) on water DC levels in the region (Fig. S2 and Fig. 4a).

Results showed that China's lake DC stocks increased by 30.6 Tg C from the 1990s to the 2010s, among which, lake DC stocks in the NWR increased by 27.1 Tg C. At the same time, the NWR had the highest DC distribution, which was mainly due to the low MAP in the region (Fig. S1 and Fig. S4). MAP and EC appeared to be the main contributors to the high inland water DC levels. The geographic and climatic characteristics of the NWR are unique compared with China's eastern region. Watershed geographic features also influence inland water carbon dynamics, steep slopes and rapid water flow in high-altitude areas enhance terrestrial organic carbon transport, while flat lowlands promote carbon burial due to prolonged water retention. Grassland is the dominant land use type in the region, which is not significantly affected by anthropogenic activities (Fig. S2). As a result, inland water in this region of China is mainly nutrient poor (Fig. S5) [16]. Song *et al.* (2018) also found that lakes on the Qinghai–Tibet Plateau have the largest overall DOC storage, accounting for ∼84.3% of China's total [17]. Additionally, reservoir construction has developed rapidly over the past 30 years, nearly doubling the storage capacity of reservoirs and more than doubling the amount of DC stored in reservoirs. Particularly in China's eastern regions, rapid urbanization, high population density, and water supply needs have accelerated dam construction (Fig. S3), especially regarding large reservoirs, leading to a surge in reservoir capacity and DC storage in these regions. River DC flux increased from 12.9 Tg C/yr in the 1990s to 13.8 Tg C/yr in the 2010s. In the NWR, annual river runoff exhibited an obvious increasing trend (Fig. 2), which reflects the expansion of the river network and the increase in flow in the region under increased precipitation, snow and ice melt, and permafrost thawing (Fig. S1). Moreover, in the context of global warming, river networks and lakes on the Qinghai–Tibet Plateau will likely expand further in the future [38]. Additionally, these have largely been unaffected by damming and other anthropogenic disturbances. The unique geographic and climatic conditions of Qinghai–Tibet Plateau water bodies have resulted in a strong potential for C storage and C neutrality.

### Water chemistry impacts

Water chemistry is the main driving mechanism (>70%) for lake and reservoir DC stocks (Fig. 4). In contrast, climatic and anthropogenic factors had a comparatively more significant influence on river DC flux. This study found that lake and reservoir DC stocks were significantly influenced by water chemistry (i.e. turbidity, pH, and nutrient status). Turbidity was the most significant variable among all influencing factors in explaining lake and reservoir DC variance (26%–31%) (*P* < 0.01; Fig. 4). Additionally, nutrient status also played a significant role in DC dynamics (*P* < 0.01; Fig. 4). In the water column, increased N and P caused phytoplankton to proliferate while increasing turbidity [46]. Turbidity regulates phytoplankton growth and photosynthetic processes through its impact on light penetration, thereby indirectly altering DOC levels in aquatic systems [47]. Structural equation modeling results showed that pH and EC exhibited a significant positive correlation with DIC concentrations in inland waters (*P* < 0.01). EC summarizes ion concentrations in the water column, particularly the level of dissolved carbonates. At the same time, in most inland waters, the carbonate system regulates pH, while pH can alter the physical and chemical environment of the water column, which in turn regulates the dynamic balance and distribution of the carbonate system within the water column [48].

Findings showed that DOC was mainly driven by nutrients. Total phosphorus (TP) on DOC path coefficients in lakes, reservoirs, and rivers were 0.43 and 0.48, respectively (*P* < 0.01). Chemometrics provides a comprehensive approach for studying relationships among ecosystem C, N, and P [49,50]. Notably, under this framework, organic matter mineralization processes lead to the release of C, N, and P into lakes. In turn, this leads to eutrophication and consequently the impairment of lake ecosystems [51]. Liu *et al.* (2022) found that the discharge of anthropogenic effluent, expressed in terms of NH_4_^+^-N, positively correlated with DOC levels in rivers [18]. Changes in climate, water temperature, and land use types can all significantly affect DOC dynamics in inland water through their influence on TP (Fig. 5). Nutrient status is significant for DOC in aquatic systems. On the one hand, it provides nutrients for phytoplankton in the water column that subsequently influences phytoplankton photosynthesis, which in turn drives primary productivity and DOC concentrations in the water column. On the other hand, nutrients in the water column are important indicators of eutrophication, and eutrophication blooms in the water column have a significant impact on the metabolism and function of aquatic systems. Phytoplankton blooms may be one potential pathway for increases in DOC, providing carbohydrates for targeting DOC [52–54].

### Implications for carbon budgets in China and throughout the world

**Figure 6.**
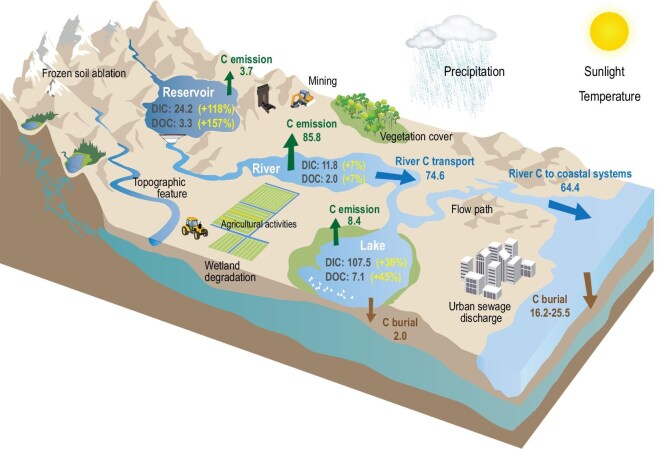
Conceptual model of driving mechanisms associated with DC storage and flux and China's inland water C budget. C storage unit: Tg C. C flux unit: Tg C/yr.

Inland waters, as important reactors and regulators of C transport, storage, and emissions, may have a significant impact on the terrestrial C balance. Globally, inland waters receive 5.1 Pg C/yr from terrestrial ecosystems, deposit 0.6 Pg C/yr in sediment, release 3.9 Pg C/yr of CO_2_ and CH_4_ to the atmosphere, and discharge 0.9 Pg C/yr to estuaries [6]. As a critical component of the global carbon cycle, China's inland waters emit ∼97.9 Tg C/yr into the atmosphere, transport ∼74.6 Tg C/yr through rivers, and export ∼64.4 Tg C/yr to coastal areas (Fig. 6). Under the enormous global climate changes taking place in conjunction with China's economy and social structure development, both inland water DC stocks and river DC flux exhibited significant increasing trends over the past 30 years. In China's inland waters, DC stocks have increased from 96.4 Tg C in the 1990s to 142 Tg C in the 2010s, while river DC flux has increased from 12.9 Tg C/yr in the 1990s to 13.8 Tg C/yr in the 2010s. In the foreseeable future, it is likely that more land in China will be converted from natural forests and grasslands to residential areas or agricultural land. This land use change will not only alter terrestrial C cycling processes but will also significantly alter inland water C cycling processes. Despite some uncertainty, our research findings suggest that inland water C dynamics play an important role in the global C balance and must therefore be accounted for in future C budget assessments. This will also help us to better understand the complex driving mechanisms associated with C as it pertains to climate and anthropogenic disturbances in China's inland waters over the past 30 years. Under a changing global background, we should pay more attention to accurately assessing the C budget of inland waters and formulating more scientific and reasonable policies for C reductions and water quality management.

## METHODS

### Study area

China's inland waters are divided into nine regions (Fig. 1): the Songhua River and Liaohe River Basin (SLR); the Haihe River Basin (HAR); the Yellow River Basin (YR); the Huaihe River Basin (HUR); the Yangtze River Basin (YZR); the Southeast River Basin (SER); the Pearl River Basin (PR); the Southwest River Basin (SWR); and the Northwest River Basin (NWR). Chinese land use data used in this study (i.e. from the 1990s, 2000s, and 2010s) were obtained from the Landsat-derived Annual Land Cover Dataset (CLCD) [55] (Fig. S2).

### Determining water volume and runoff

#### Lake and reservoir volume

The data related to water-surface changes during the1990s to the 2010s were obtained from the China lake dataset (1960s–2020) issued by the National Tibetan Plateau/Third Pole Environment Data Center (http://data.tpdc.ac.cn). This dataset includes lakes in China with a surface area >1 km^2^, which account for ∼92% of its total lake surface area. Thus, this dataset is a reasonable representation of overall lake conditions throughout the country [56]. The total lake capacity was calculated using empirical equations according to Yang and Lu's method, where lake volume is primarily based on surface area. The equations used for lakes were calculated via the following Equation (1) [56]:


(1) \begin{eqnarray*}
{\mathrm{\ Lakes\!:\ }}V = 1.2601{A}^{1.1726}( {{R}^2 = 0.7711} ),
\end{eqnarray*}

where *V* is the lake volume in 10^6^ m^3^ and *A* is the surface area in km^2^.

Data on distribution and reservoir dam capacity from the 1990s to the 2010s are summarized by the *China Water Conservancy Yearbook* (Ministry of Water Resources [MWR], the People's Republic of China).

#### River runoff

We obtained annual runoff data from the 1990s to the 2010s from the China Natural Runoff Dataset version 1.0 (CNRD v1.0), which uses previously constructed 0.25° × 0.25° monthly runoff datasets from a calibrated land surface model [57]. CNRD v1.0 was generated using the Variable Infiltration Capacity (VIC) macroscale land-surface model and coupled Lohmann routing model. A comprehensive parameter uncertainty analysis framework incorporating parameter sensitivity analysis, optimization, and regionalization with 200 natural or near-natural gauge catchments was used to train the VIC model.

### Carbon data acquisition

In total, we obtained 1997 DIC data and 1978 DOC data through field measurements, collection from published literature, and model estimation. We collected water samples from 2017 to 2022 from 156 sampling sites from the Yangtze River, Poyang Lake, and the Qinghai–Tibet Plateau. Water samples were filtered through 0.45 µm pore size organic membrane filters, which were first treated in a water bath (80°C) for 8 hours prior to filtering. DOC and DIC levels were determined with the Vario TOC Analyzer (Elementar, Germany).

Although our sample site number was relatively large (i.e. 156 sites), gaps remained whereby representative DC concentrations are not known (Fig. 1a). Accordingly, we adopted the method proposed by Sobek *et al.* (2007) to assemble data on inland water DC concentrations across China from published literature and national environmental surveys [58]. In cases where the total organic carbon (TOC) concentration was provided, it was converted to DOC by multiplying the value by 0.9 [31,59]. Additionally, for certain inland water systems where DOC concentrations were not available, we used the surrogate parameter chemical oxygen demand (COD) as an alternative [60,61]. We obtained daily COD data from 1452 stations nationwide between June 2021 and May 2022 from the China National Environmental Monitoring Centre (CNEMC). To avoid seasonal differences, daily data from each station were averaged throughout the year. The relationship between COD and DOC in various inland water types (i.e. lakes, reservoirs, and rivers) was based on findings published by Song *et al.* (2018) and Liu *et al.* (2022) [17,^18^].

When DIC concentrations were not available for certain inland water bodies, we used the CO2SYSv3 program (run in MATLAB) to calculate DIC concentrations based on total alkalinity, pH, and water temperature (WT) [62–64]. Daily pH and water temperature data from the 1416 stations (nationwide) between June 2021 and May 2022 were obtained from the CNEMC. Alkalinity was estimated using five indicators, including pH, dissolved oxygen (DO), electrical conductivity (EC), total nitrogen (TN), and total phosphorus (TP), from CNEMC in conjunction with a machine learning model, namely, Random Forest with bagged trees (RF-BAT), which was trained using the GLObal RIver CHemistry database (GLORICH) and validated via *in situ* measurements from typical lakes in China. Water overestimations under low pH (<6.5) and alkalinity (<1000 µeq/L) conditions were corrected with an empirical model developed by Liu *et al.* (2020) [65]. See Sun *et al.* (2023) for more details on calculations and corrections [66]. To take into account seasonal differences, daily data were averaged at each site throughout the year. Additionally, some C flux data in Fig. 6 is derived from [38,67–70].

### Water carbon storage and flux quantification methods

Lake and reservoir DIC and DOC storage in the nine aforementioned river basins was derived from Equation (2):


(2) \begin{eqnarray*}
S = C \times V,
\end{eqnarray*}

where *S* is the C storage of lakes and reservoirs in a river basin; *C* is the C concentration that is calculated by averaging multi-source data from lakes and reservoirs in a river basin; *V* represents the sum of volume of lakes and reservoirs in a river basin.

River DIC and DOC flux was derived from Equation (3):


(3) \begin{eqnarray*}
F = C \times R,
\end{eqnarray*}

where *F* is the C flux of rivers in a river basin; *C* is the C concentration that is the average of multi-source data from rivers in a river basin; *R* represents the total runoff of rivers in a river basin.

### Statistics

Standard deviations (SDs) of inland water DC storage and flux were calculated via Origin 9.1 software. Analysis of variance (ANOVA) tests were used in this study to determine whether the difference between two grouped samples was significant or not. Linear regression was performed to explore changes in water volume. We applied ArcGIS 10.4 for statistics, calculations, and mapping of the study data. The average value of each data point in each three-level watershed was used to explore the impact of various influencing factors on spatiotemporal DC patterns. The contributions of climate and anthropogenic factors to spatiotemporal inland water DC storage and flux patterns in different river basins were analyzed via generalized linear mixed models (GLMM) in RStudio. The ‘glmm.hp’ and ‘lme4’ packages were used to build the GLMM and perform variance decomposition as well as to analyze the relative contributions of influencing factors [71]. The contributions of different influencing factors to spatiotemporal inland water DC storage and flux patterns were analyzed through random forest regression models in RStudio. Partial least squares structural equation modeling (PLS-SEM) was conducted in RStudio, which was used to evaluate the direct and indirect effects of various influencing factors on China's inland water DC patterns.

### Uncertainty

Uncertainty in DC storage estimations mainly derives from water volume estimations and DC concentration statistics. Errors in lake water volume estimations mainly derived from unpredictability in water area extraction, inaccuracy in boundary water divisions, and uncertainty in water depth. The lake area data used in this study only included lakes with an area ≥1 km^2^, accounting for ∼92% of China's total lake area, which reflects the country's overall lake conditions [56]. Uncertainty also derives from water area statistics of the nine aforementioned river basins in China. Some rivers, lakes, and reservoirs extend into multiple areas, which can create some errors when assigning the water area of each basin. Additionally, lake depth uncertainty is responsible for part of the error, while lake water volume is mainly calculated based on surface area (using empirical Equation (1); *R*^2^ = 0.77). Using remote sensing to establish water depth remains very challenging and is likely to be subject to significant uncertainty [72]. However, reservoir water volume data are relatively accurate, causing less uncertainty.

Uncertainty in C concentrations derives from DIC concentrations estimated from alkalinity data and DOC concentrations derived from TOC and COD concentrations. First, for some inland water bodies where DIC concentrations were not available, we first used the CO2SYS program (run in MATLAB) to calculate DIC concentrations based on total alkalinity, pH, water temperature, and atmospheric pressure [62,63]. To account for seasonal variations, averages of daily data were compiled from national water quality sites throughout the year. In recent years, this method has been widely used in many studies to estimate indicators in carbonate equilibria systems, and its reliability has been confirmed [73]. Second, for some inland water bodies where DOC concentrations could not be collected, we used TOC and COD concentrations for our calculation, multiplying TOC by 0.9 to convert to DOC [31,59] as well as the empirical relationship between COD and DOC in different water bodies. The COD of lakes and reservoirs can explain 82% of DOC variation, while the COD in rivers can explain 55% of DOC variation [16,17]. Additionally, this study disregarded DC concentration changes with water depth, which could also cause some uncertainty. However, a few studies have shown that most water bodies undergo little change in DC concentrations in the vertical water column [16,74]. Therefore, for lakes and reservoirs, uncertainty caused by DC variation along the depth profile is negligible, especially considering that China has no extremely deep lakes or reservoirs.

## Supplementary Material

nwaf229_Supplemental_File
