# Supplementary material for: Dissolved carbon storage and flux dynamics in China's inland waters over the past 30 years
Source: Natl Sci Rev. 2025 Jun 2;12(8):nwaf229. doi: 10.1093/nsr/nwaf229 (PMC12243852; doi:10.1093/nsr/nwaf229)
Supplement: nwaf229_Supplemental_File [file nwaf229_supplemental_file.docx]

**Supplementary materials**

**Text S1**

**Inland-water C concentrations over China**

The DIC and DOC concentrations in inland waters of China varied considerably, while the DC concentrations in lakes, reservoirs and rivers also showed different characteristics. Overall, DIC concentrations in inland waters of China were larger in the NWR, SWR, and YR, and smaller in the YZR and SER (Fig. S4). DOC concentrations were larger in the SLR and NWR (especially in the Tibetan Plateau region), and smaller in the SER. Among the different types of water bodies, DIC (123.12 mg/L) and DOC (13.6 mg/L) concentrations were much higher in lakes than in rivers and reservoirs, and DIC concentrations were highest in lakes in the NWR, with a mean value of 544.38 mg/L, and lower in the YZR and SER (Fig. S4). DOC concentrations were higher in lakes in the NWR (26.2 mg/L) and SLR (31.91 mg/L) and lower in the SER (Fig. S4). The DIC concentrations in reservoirs showed a less homogeneous character, with those in the PR (53.83 mg/L) being significantly higher than in the other basins, while those in the SER and SLR were less than 4 mg/L). Among the rivers, the DIC concentration levels were higher in the NWR and the YR, and lower in the SER, while the difference in DOC concentration among the rivers in the basins was small, with a mean value of 4.95 mg/L (Fig. S4).

**Water quality data acquisition**

The daily data of water quality parameters (WT, pH, DO, EC, TN, and TP) for 1452 stations nationwide from June 2021 to May 2022 were obtained from the China National Environmental Monitoring Centre (CNEMC). To avoid seasonal differences, we averaged the daily data for each station throughout the year. In addition, from 2017 to 2022, these water quality parameters were collected from the main stream of the Yangtze River, Poyang Lake, and the Tibetan Plateau. The Ultrameter Model 6P multimeter water quality instrument (MYRON, the United States of America) was used to measure WT, EC, and pH in situ. The portable fluorescence dissolved oxygen meter (Yellow Springs Instruments, the United States of America) was used to measure DO in situ. Water samples were filtered through 0.45‐μm organic microporous membrane filters, which were first treated in a water bath (80°C) for 8 h prior to filtering. TN and TP concentration was tested using a digestion method that employed a segmented continuous flow analyzer (Futura, France).

**Other data source**

Temperature and precipitation data were obtained from the China meteorological forcing dataset (1979-2018) (<https://poles.tpdc.ac.cn/zh-hans/>) (He et al., 2020). Land use data were obtained from the 30 m annual China land cover dataset (CLCD) from 1990 to 2021 (Yang and Huang, 2021). The boundary data of nine river basins in China comes from the Resource and Environmental Science Data Platform (https://www.resdc.cn/).Data on the number of dams and reservoir area retrieved from the Global Reservoir and Dam (GRanD v1.1; http://www.gwsp.org/products/grand-database.html) database. There are 773 reservoirs in China with a total reservoir capacity of more than 0.1 km^2^. Reservoir capacity is mainly calculated based on surface area according to the equation: C = 25.841A^1.05^ (C is reservoir storage volume for individual reservoirs in 10^6^ m^3^ and A is the surface area in km^2^, R^2^=0.9097) (Yang and Lu, 2014).


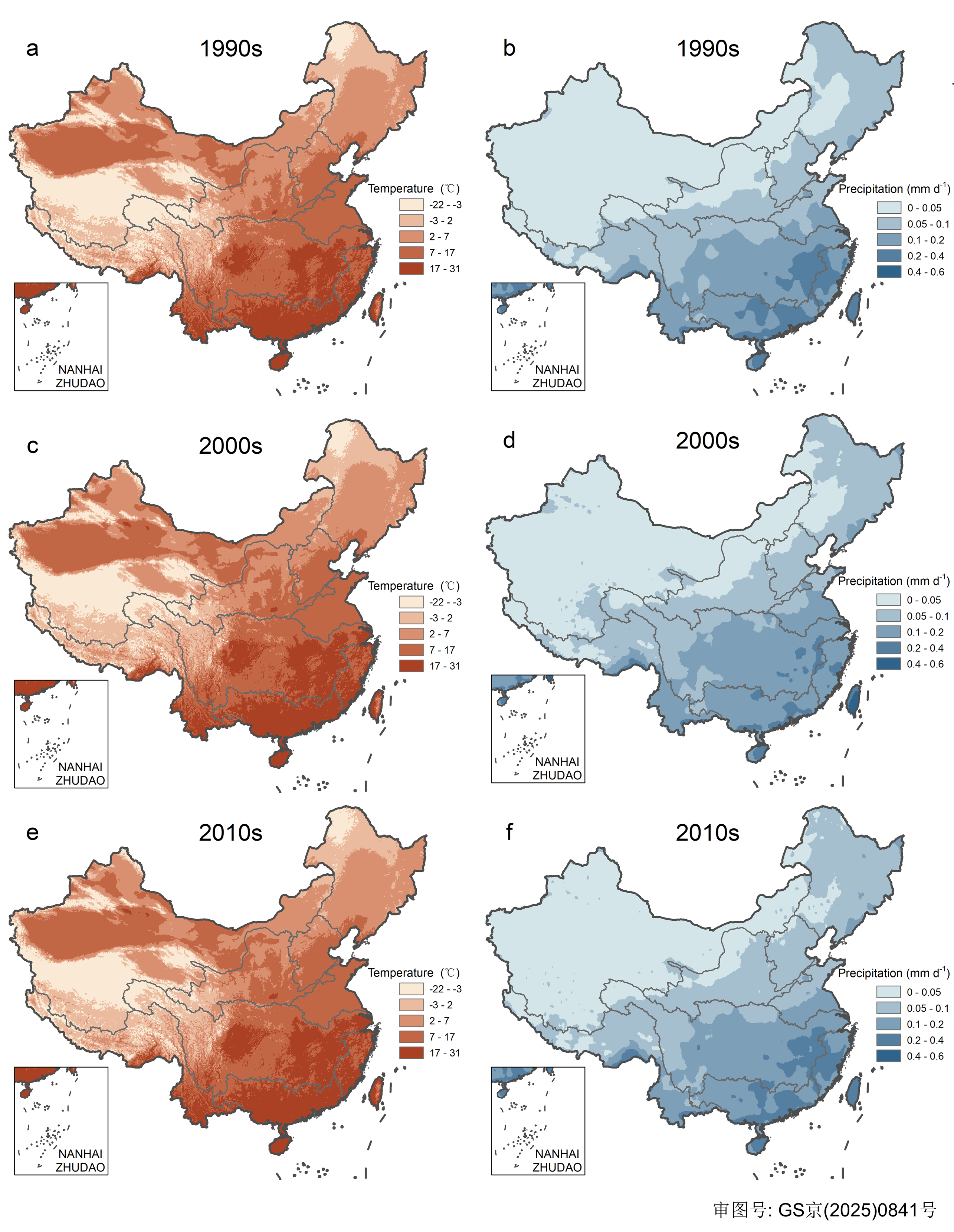


Fig. S1.

Annual mean temperature and precipitation distribution in China from 1990s to 2010s.


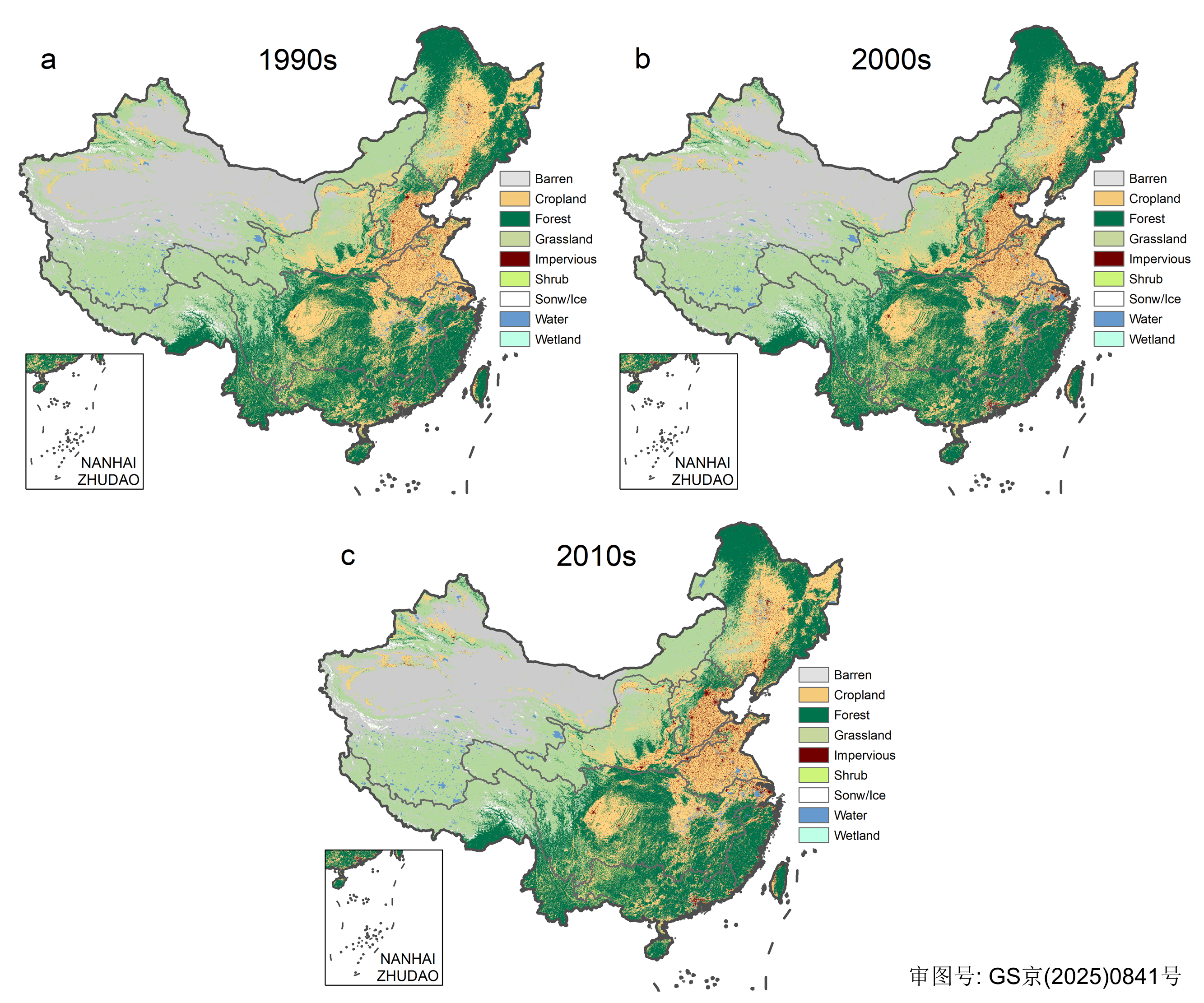


Fig. S2.

Distribution characteristics of land cover in China from 1990s to 2010s.


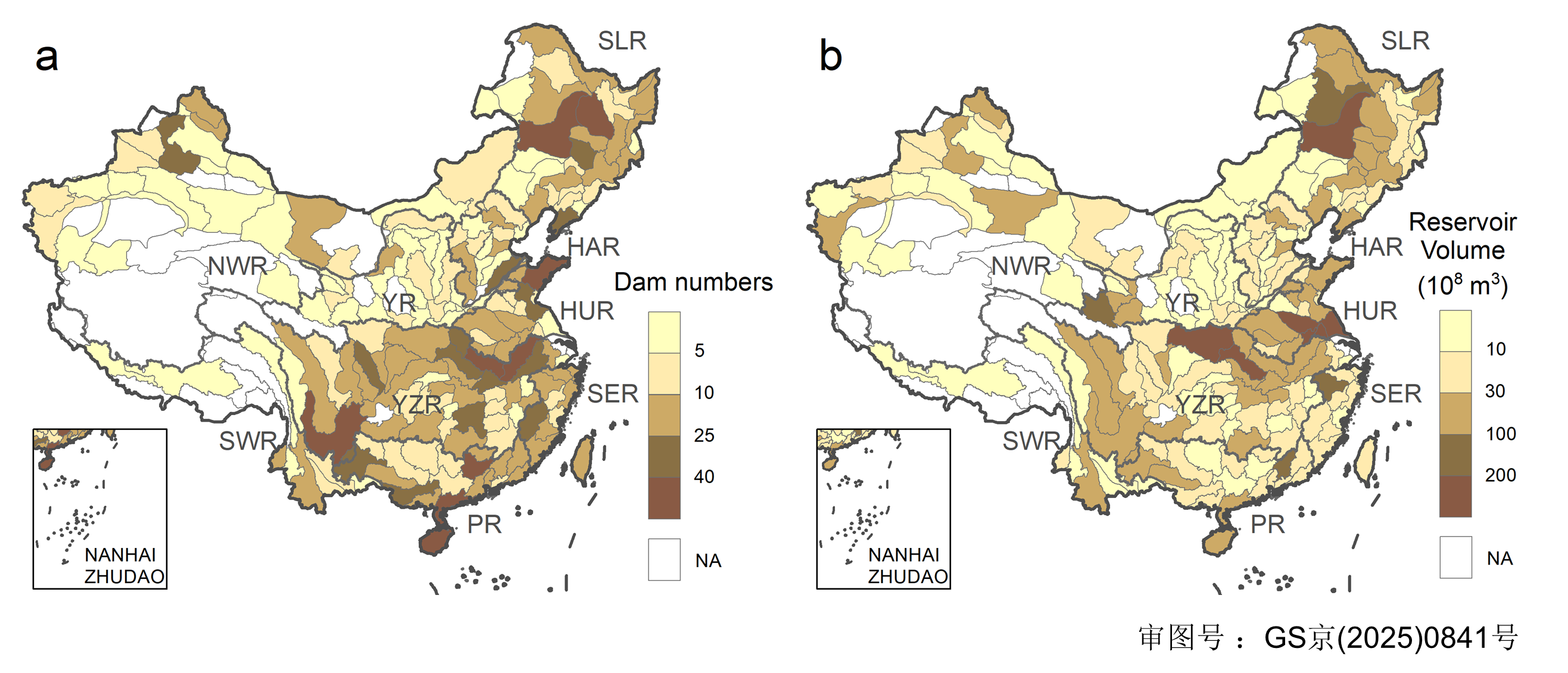


Fig. S3.

Number of dams (a) and reservoir volume (b) in China; data on the number of dams and reservoir area retrieved from the Global Reservoir and Dam (GRanD v1.1; http://www.gwsp.org/products/grand-database.html) database. There are 773 reservoirs in China with a total reservoir capacity of more than 0.1 km^2^. Reservoir capacity is mainly calculated based on surface area according to the equation: C = 25.841A^1.05^ (C is reservoir storage volume for individual reservoirs in 10^6^ m^3^ and A is the surface area in km^2^, R^2^=0.9097) (78).


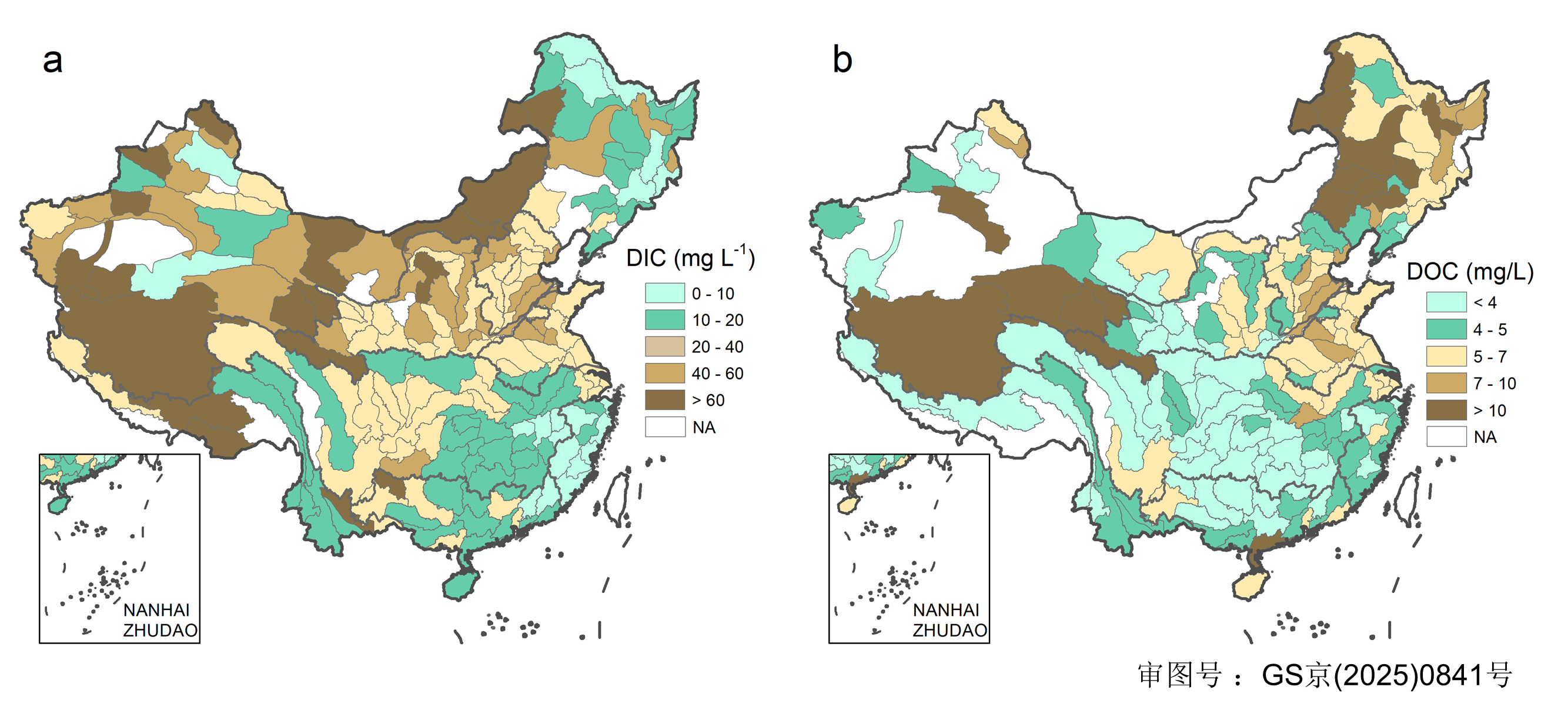


Fig. S4.

Distribution characteristics of DC concentrations in inland waters in China.


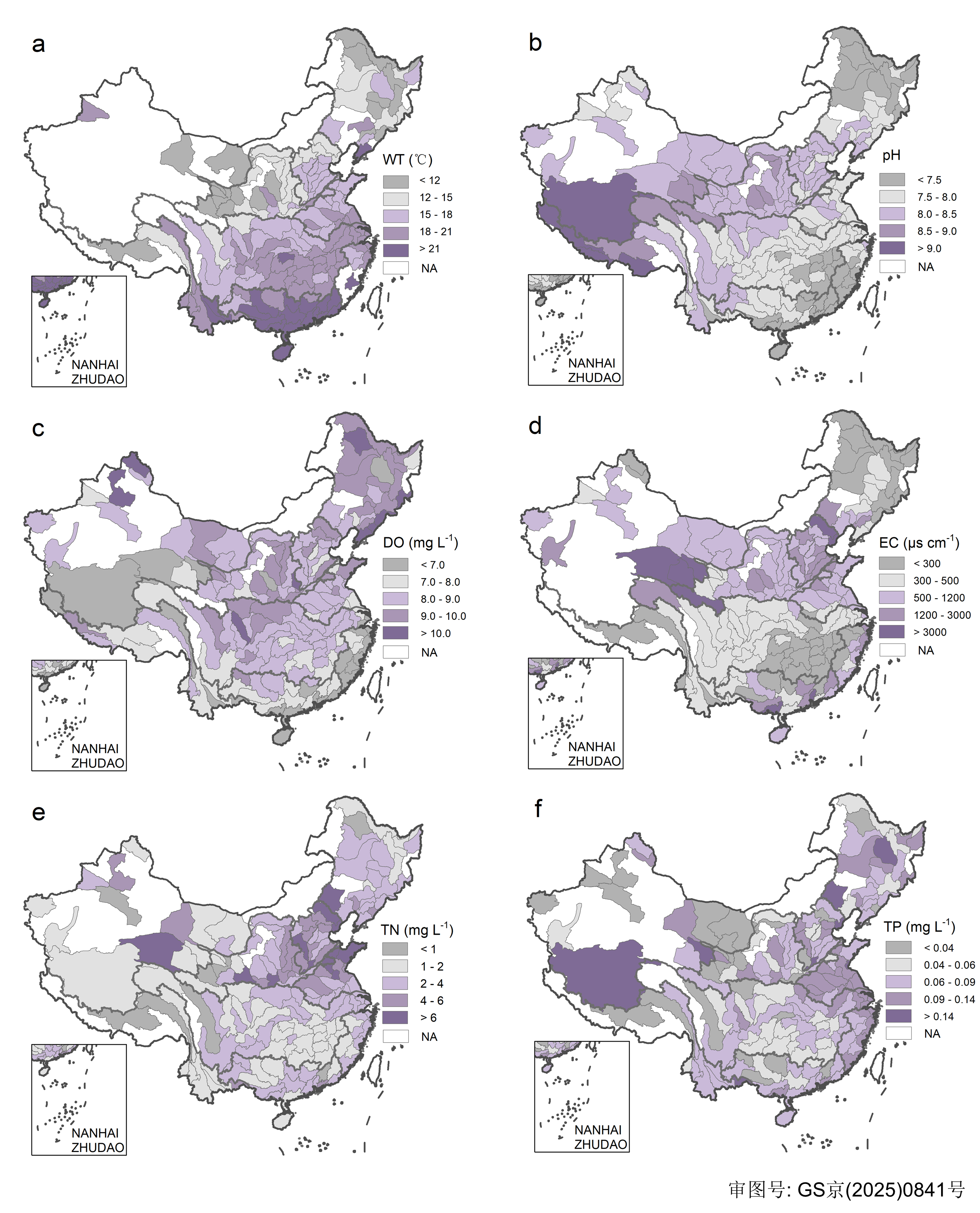


Fig. S5.

Distribution characteristics of water quality parameters of inland waters in China. WT: water temperature, DO: dissolved oxygen, EC: electrical conductivity, TN: total nitrogen, and TP: total phosphorus.

**References**

He, J., Yang, K., Tang, W., Lu, H., Qin, J., Chen, Y., & Li, X. (2020). The first high-resolution meteorological forcing dataset for land process studies over China. Scientific data, 7(1), 25.

Yang, J., & Huang, X. (2021). The 30 m annual land cover dataset and its dynamics in China from 1990 to 2019. Earth System Science Data, 13(8), 3907-3925.

Yang, X., & Lu, X. (2014). Drastic change in China's lakes and reservoirs over the past decades. Scientific reports, 4(1), 6041.
